# Enhanced Tribological and Electrical Performance of Graphene-Coated Polyetheretherketone Nanocomposites

**DOI:** 10.3390/polym17060721

**Published:** 2025-03-09

**Authors:** Pyoung-Chan Lee, Seo-Hwa Hong, Jung-Hoon Kim, Jae-Young Seo, Youn-Ki Ko, Jin-Uk Ha, Sun-Kyoung Jeoung, Myeong-Gi Kim, Beom-Gon Cho

**Affiliations:** 1 Chassis & Materials Research Laboratory, Korea Automotive Technology Institute, 303 Pungse-ro, Pungse-myeon, Dongnam-gu, Cheonan-si 31214, Chungcheongnam-do, Republic of Korea; pclee@katech.re.kr (P.-C.L.); shhong1@katech.re.kr (S.-H.H.); ykko@katech.re.kr (Y.-K.K.); juha@katech.re.kr (J.-U.H.); skjeong@katech.re.kr (S.-K.J.); 2 R&D Center, BESTGRAPHENE Co., Ltd., Yeoju-si 12616, Gyeonggi-do, Republic of Korea; run8827@best-graphene.com (J.-H.K.); jyseo@best-graphene.com (J.-Y.S.); 3 Department of Polymer Science and Engineering, Kumoh National Institute of Technology, 61 Daehak-ro, Gumi-si 39177, Gyeongbuk, Republic of Korea

**Keywords:** polyetheretherketone (PEEK), chemical modified graphene (CMG), nanocomposites, friction, electric resistance

## Abstract

Polyetheretherketone (PEEK) is widely used across various industries due to its high thermal stability, chemical resistance, and superior mechanical properties. However, its tribological and electrical properties require enhancement for advanced applications. This study investigates the effect of graphene coating on PEEK microspheres to improve their performance. Functionalized graphene oxide (CMG+) and graphene nanoplatelets (GnPs) were introduced onto the PEEK surface via an electrostatic self-adsorption process, followed by high-speed mixing and hot-pressing to fabricate PEEK–graphene nanocomposites. The structural, thermal, tribological, and electrical properties of the composites were systematically analyzed. The results show that graphene acts as a nucleating agent, enhancing the crystallinity of the nanocomposites. Tribological tests indicate that CMG+ significantly reduces the friction coefficient, with CMG1.0 and CMG2.0 samples showing friction reductions of 54% and 63%, respectively, compared to pure PEEK. Moreover, electrical property evaluations reveal that surface resistance decreases with increasing graphene content, achieving optimal conductivity at 1.0 wt.% CMG+ and further enhancement with the addition of GnPs. These findings demonstrate that the functionalized graphene-coated PEEK microspheres exhibit superior tribological and electrical performance due to nanoscale interactions, making them suitable for electrostatically dissipative and wear-resistant applications.

## 1. Introduction

Polyetheretherketone (PEEK) belongs to the polyaryletherketone (PAEK) family, which includes several high-performance thermoplastics such as polyetherketone (PEK), and polyetherketoneketone (PEKK). These polymers share exceptional thermal stability, chemical resistance, and mechanical properties, making them highly desirable for use in aerospace, automotive, and biomedical industries. Among them, PEEK has gained particular attention due to its balanced combination of high strength, wear resistance, and processability. These characteristics are attributed to its aromatic structure and semi-crystalline nature, which are influenced by its thermomechanical processing history. Owing to its durability and high-temperature resistance, PEEK is widely used in automotive, medical, and aerospace industries as a substitute for metal components [1,2,3,4,5,6,7,8,9,10,11,12,13,14,15,16].

In industrial production, semi-finished products, including sheets, blocks, and rods, are manufactured in pre-final stages. Several polymer-based semi-finished products are fiber-reinforced plastics (FRPs) with thermoset matrices such as epoxy and unsaturated polyester, typically produced by pultrusion. When high mechanical strength is unnecessary, thermoplastic-based semi-finished products such as PEEK are often preferred. The chemical resistance and dimensional stability of PEEK make it well suited for semiconductor and display components, where dimensional precision is essential. For insulating polymer components, maintaining a surface resistance between 10^4^ and 10^8^ ohms/sq can mitigate static electricity. To achieve this, conductive additives such as carbon black, carbon fibers, carbon nanotubes, and graphene are commonly added to composites [17,18,19].

Various fillers are added to polymer matrices to enhance their properties, significantly affecting their mechanical, chemical, electrical, thermal, and tribological performances [4,5,6,7,8,9,10,11,12]. The effect of fillers on these properties depends on factors such as composition, shape, and size. Common strategies for improving PEEK include the addition of carbon-based fillers [4,5,6], ceramic fillers [7,8], glass and carbon fibers [9,10,11,12], and solid lubricants [13,14]. Studies have shown that nanoscale fillers improve wear resistance and mechanical properties, with the filler shape playing an essential role in frictional behavior [2].

Graphene, a two-dimensional monolayer of graphite, has attracted attention as a filler for its outstanding electrical, mechanical, wear-resistant, and thermal properties [20,21]. Owing to its high elastic modulus and tensile strength, graphene enhances the stiffness and mechanical integrity of composites [1,15,16,22,23,24]. Its large surface area provides a broad interface for polymer matrices, which improves load transfer and overall composite strength. Furthermore, graphene’s two-dimensional structure can function as a lubricant additive, solid lubricant, or sliding coating [1,22,23,24,25,26,27]. Previous studies have investigated how graphene fillers affect the thermal stability and tribological and mechanical properties of polymers, such as Nylon 6 [27], Nylon 46 [22], polyacrylonitrile [28], polyimide [29], and PEEK [1,2,23,24]. Puértolas et al. [1] developed PEEK–graphene nanoplatelets (GnPs) through solvent-free melt blending and injection molding. Compared to conventional PEEK, these nanocomposites demonstrated enhanced surface hardness and a lower friction coefficient [22]. However, the high concentration of GnPs required (1–10 wt.%) and the challenges of achieving uniform dispersion through melt blending alone limited the full benefits of graphene [22].

In this study, we developed an efficient method to uniformly disperse graphene to enhance the tribological and electrical properties of PEEK. PEEK–graphene nanocomposites were prepared through controlled interactions between chemically modified graphene (CMG) and PEEK. CMG was designed to promote chemical interactions with the polar groups of PEEK. We analyzed the effects of graphene content on the melting behavior, friction, and electrical properties of PEEK–graphene nanocomposites with the goal of enhancing performance while ensuring uniform dispersion.

## 2. Materials and Methods

### 2.1. Materials

Ultrafine PEEK powder (880UFP grade) was sourced from Solvay Co. (Brussels, Belgium). Graphite flakes, with a thickness of 150 μm and specifically designed for graphene applications, were obtained from Graphene Supermarket (Ronkonkoma, NY, USA). Chemicals including 4,4′-oxydianiline (>97%, Sigma-Aldrich, St. Louis, MO, USA), N,N-dimethylformamide (DMF, >99.5%, TCI, Tokyo, Japan), acetone (>99.5%, TCI, Tokyo, Japan), and 1-methyl-2-pyrrolidinone (NMP, >99%, TCI, Tokyo, Japan) were used without additional pre-treatment. Expanded graphite (ES 100, Samjungcng Co., Gyeongsan, Republic of Korea) was used to produce graphene nanoplatelets (GnPs).

### 2.2. Preparation of Nanofiller

The CMG was prepared to facilitate molecular-level chemical interactions with PEEK. Accordingly, a graphene oxide (GO) containing hydroxyl, carboxyl, and epoxide groups was prepared to fabricate the CMG as outlined in previous studies [15,30,31].

A graphene surface modification reaction was conducted using 4,4′-oxydianiline to obtain CMG colloids capable of interacting with PEEK [22,31]. A solution of 0.1 wt.% of GO (500 mL) was prepared via ultrasonication for at least 2 h. Subsequently, 4,4′-oxydianiline (20 g) was added to 500 mL of DMF, and the mixture was stirred at 90 °C for 40 h. The reaction product was cooled to 20–25 °C and then subjected to centrifugation to obtain the precipitate. The precipitate was washed three to five times with acetone and filtered. The resulting graphene layers were immediately placed in an ethanol bath. The homogenization process facilitated the protonation of amine groups, leading to the formation of positively charged CMG (CMG⁺). As a result, a highly dispersive functionalized graphene colloid was generated, with solvation effects in the ethanol medium and the possible presence of ethanol-derived counterions (e.g., ethoxide, CH_3_CH_2_O⁻). The graphene content was adjusted to 1 wt.% by controlling the amount of solvent.

The GnPs were fabricated with liquid exfoliation as follows: the NMP solution was ball-milled at 50 rpm for 24 h with 10 wt.% expanded graphite (ES 100 grade) for expansion treatment. Subsequently, the samples were subjected to primary pulverization and homogenization using a homogenizer (15,000 rpm, 1 h), followed by high-pressure homogenization at 1500 bar for 10 passes. Finally, a 10 wt.% GnP with NMP dispersion was prepared by passing the sample through a 100 μm filter in a pressure filtration system and homogenizing with a homogenizer.

### 2.3. Preparation of Nanocomposites

**Figure 1:**
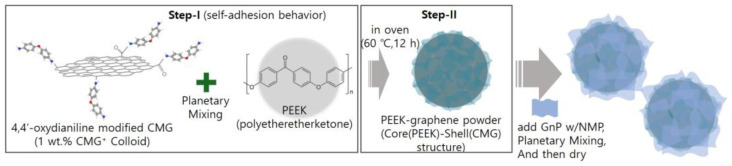
Schematic illustration of the fabrication process for PEEK–graphene nanohybrid materials. **Step I:** Chemically modified graphene (CMG⁺) is mixed with PEEK via planetary mixing, forming a core–shell (PEEK-core/CMG-shell) structure through self-adhesion. **Step II:** The mixture is dried at 60 °C for 12 h, followed by the addition of graphene nanoplatelets (GnPs) dispersed in NMP, further mixed and dried.

As shown in Figure 1, in step 1, the PEEK powder was mixed with 1.0 wt.% CMG^+^ colloid. The graphene content was set to 0.1, 0.3, and 0.5 wt.%. The mixture was subjected to high-speed revolution and rotation using a Thinky mixer at revolution and rotation speeds of 2000 and 800 rpm, respectively, with each cycle lasting 3 min. This process was repeated three times. The samples produced using 0.1, 0.3, and 0.5 wt.% graphene were named CMG0.1, CMG0.3, and CMG0.5, respectively. In step 2, PEEK–graphene powder with a PEEK(core)–CMG(shell) structure was fabricated by drying the as-prepared samples in a vacuum oven at 60 °C for 12 h under a pressure of 0.1 bar. Steps 1 and 2 were conducted once to produce CMG0.5. CMG1.0 and CMG2.0 were manufactured by repeating steps 1 and 2 two or four times, respectively. The hybrid sample CMG1.0/GnP1.0 was prepared by adding 10 g of a 10 wt.% GnP with NMP dispersion to 100 g of CMG1.0 powder. The mixture was then subjected to high-speed revolution and rotation mixing using a Thinky mixer at revolution and rotation speeds of 2000 and 800 rpm, respectively, for 3 min. The cycles were repeated three times. Finally, the CMG1.0/GnP1.0 sample was obtained by drying the product in a vacuum oven at 120 °C for 12 h. To analyze the properties of PEEK–graphene powder, specimens were fabricated via hot press (Qmesys, Uiwang, Republic of Korea). After placing the powder into a mold, it was subjected to a pressure of 5 bar for 30 min at a temperature of 380 °C.

### 2.4. Characterization

Elemental analysis (EA, FlashSmart, Thermo Fisher Scientific, Waltham, MA, USA) was conducted to examine the chemical modifications of GO and graphene operating in CHNS mode. The morphologies of CMG^+^ and GnPs were analyzed using atomic force microscopy (AFM, FX40, Park Systems, Suwon, Republic of Korea) and scanning electron microscopy (SEM, VEGA, TESCAN, Brno, Czech Republic) with 20 keV and the SE mode, respectively. The graphene additives were analyzed using zeta potential measurements (LITESIZER 500, Anton Paar, Graz, Austria).

The PEEK–graphene powder was analyzed by SEM. The particle size distributions in pristine PEEK and PEEK–graphene powder were measured using a laser diffraction particle size analyzer (Mastersizer, Malvern Panalytical, Worcestershire, UK) in the range of 10 nm–3500 μm. The melt flow index (MI) of PEEK–graphene powder was analyzed using a melt flow indexer (MI-2, GöTTFERT, Buchen, Germany) at a temperature of 380 °C and load of 5.2 kg.

The effect of graphene content on crystallization was evaluated using a polarization microscope (VHX-7000, Keyence, Osaka, Japan) in conjunction with a hot stage (LTS420, Linkam Scientific Instruments, Salfords, UK). The powder was heated at a rate of 50 °C/min until 400 °C, after which the temperature was held at 400 °C for 5 min to melt the powder. Subsequently, the sample was cooled at a rate of 10 °C/min until reaching 30 °C, resulting in the formation of crystals. Furthermore, the crystallinity of the PEEK–graphene powder was analyzed via differential scanning calorimetry (DSC, DSC4000, PerkinElmer, MA, USA) at a heating rate of 10 °C/min in the temperature range of 50–400 °C. To ensure accurate thermal characterization, **DSC measurements were performed** after sealing the samples in an aluminum pan under a nitrogen atmosphere. The melting temperature (T_m_) was defined as the temperature corresponding to the maximum endothermic peak observed during the heating scan, whereas the crystallization temperature (T_c_) was defined as the temperature corresponding to the minimum exothermic peak observed during the cooling scan. The degree of crystallinity of PEEK in the PEEK–graphene nanocomposites, X_c_, was obtained by dividing the corrected melting enthalpy by the melting enthalpy of a 100% crystalline PEEK (130 J/g) [23]. Thermogravimetric analysis (TGA) was performed using a TGA4000 instrument (Perkin Elmer, Waltham, MA, USA). The analysis was conducted under a nitrogen atmosphere at a heating rate of 10 °C/min, with a gas flow rate of 20 mL/min.

The friction properties of PEEK–graphene nanocomposites were determined using a pin-on-disk tester (THT, Anton Paar, Graz, Austria). This test was conducted using a 10 N test load at a motor speed of 400 rpm under an ambient temperature of 25 °C. A stainless steel ball with a diameter of 6 mm was used. The surface resistance of the PEEK–graphene nanocomposites was measured using a resistance meter (Trek 152, Advanced Energy, Denver, CO, USA). All physical property evaluations were conducted five times (n = 5) to ensure reproducibility and minimize experimental variability.

## 3. Results

### 3.1. Characterization of GnPs and CMG^+^

EA was used to elucidate the molecular and structural characteristics of the synthesized GnPs and CMG^+^. As shown in Table 1 and Table S1, the measured wt.% values were then normalized to obtain normalized wt.%, ensuring that the sum of all detected elements equaled 100%. Atomic ratios (at.%) were calculated by dividing the normalized wt.% by the atomic mass of each element. A hydrogen-excluded atomic ratio (%) was separately presented, allowing a direct comparison of C, O, and N contents. This is particularly useful for cases where hydrogen is not the primary focus of compositional analysis. Table 1 shows the compositions of C, O, and N in GO, CMG^+^, and GnPs. CMG^+^ had C and N contents of 80.10 at.% and 10.05 at.%, respectively, indicating successful functionalization through the reduction of GO and substitution with 4,4′-oxydianiline.

**Figure 2:**
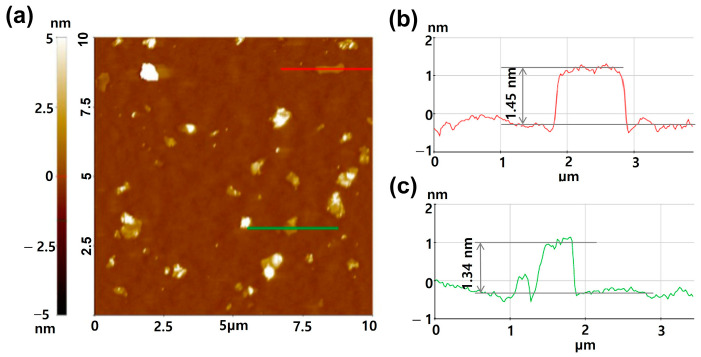
(**a**) AFM non-contact mode image of CMG^+^ on a Si wafer substrate. (**b**) Height profile corresponding to the red line in (**a**), indicating a thickness of approximately 1.45 nm; (**c**) height profile corresponding to the green line in (**a**), showing a thickness of 1.34 nm.

The change in the thickness of the CMG^+^ sheet was measured using AFM. Figure 2 shows AFM images and height profiles of the CMG^+^ sheet. The thickness of the CMG^+^ sheets varied between 1.45 and 1.34 nm. These values are consistent with the thickness of single-layer graphene on Si wafer substrates.

Figure S1 shows the SEM micrographs of the GnPs. Compared with CMG^+^ [20], the GnPs were thicker, possibly owing to slight stacking or agglomeration between the GnP sheets. CMG^+^ was dispersed as a monolayer in the colloid because of the strong electrostatic repulsion between the graphene sheets [22]. However, the GnPs produced through swelling and mechanical delamination were in the form of flakes with thicknesses of 20–30 nm, indicating graphene sheet stacking.

### 3.2. Characterization of PEEK-Graphene Nanocomposites

#### 3.2.1. Structural and Surface Analysis

**Figure 3:**
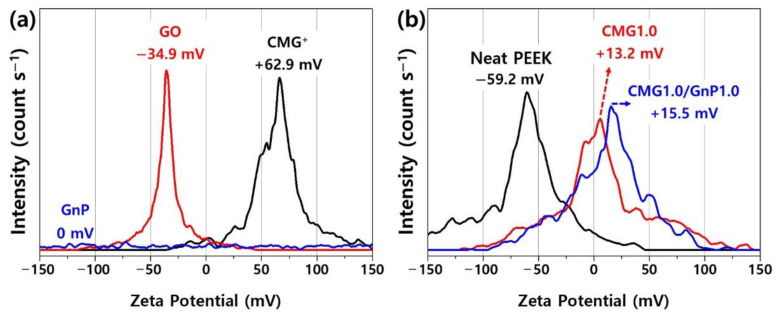
Zeta potentials of (**a**) GO, CMG^+^, GnP, (**b**) pristine PEEK, CMG1.0, and CMG1.0/GnP1.0.

Figure 3 shows the zeta potentials of GO, CMG^+^, GnP, PEEK, and PEEK–graphene nanocomposites. GnP, GO, and CMG exhibited a zeta potential of 0, −34.9, +62.9 mV, respectively. The zeta potential of PEEK was influenced by the presence of ether and ketone groups in its polymer chain and was measured to be −59.2 mV, indicating a highly negative surface charge. These results suggest that PEEK–graphene nanocomposites can be easily and rapidly fabricated using CMG^+^ owing to the strong electric charge and spontaneous bonding induced by the electrostatic adhesion of the graphene sheets inside the nanocomposites. As shown in Figure 3b, CMG1.0 exhibited a zeta potential of +13.2 mV. This indicated the successful adsorption of CMG^+^ and PEEK, which have positive and negative surface charges, respectively, onto the PEEK surface via electrostatic forces and ion binding. The hybrid CMG1.0/GnP1.0 sample consisted of GnPs that were physically adsorbed onto the surface of the CMG1.0 powder. This adsorption was induced through van der Waals forces between CMG^+^ and GnPs, as well as through π-π stacking. Therefore, its zeta potential was close to that of CMG1.0, specifically +15.5 mV. During the injection or extrusion molding of the PEEK–graphene nanocomposite, CMG^+^ formed a three-dimensional network through ion bonding with the PEEK polymer chain. The GnPs were expected to easily detach from the surface of CMG^+^ and disperse freely into the graphene sheets inside the nanocomposites.

**Figure 4:**
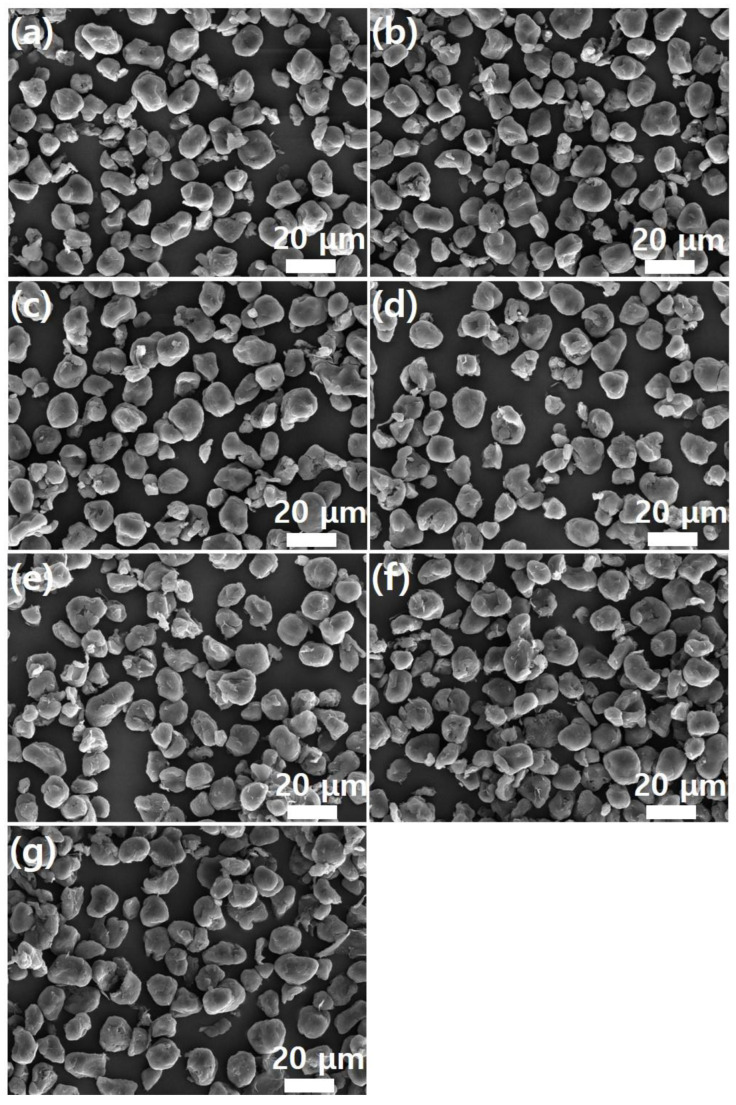
SEM images (low magnification) of PEEK–graphene powder for various graphene contents: (**a**) neat PEEK, (**b**) CMG0.1, (**c**) CMG0.3, (**d**) CMG0.5, (**e**) CMG1.0, (**f**) CMG2.0, and (**g**) CMG1.0/GnP1.0.

**Figure 5:**
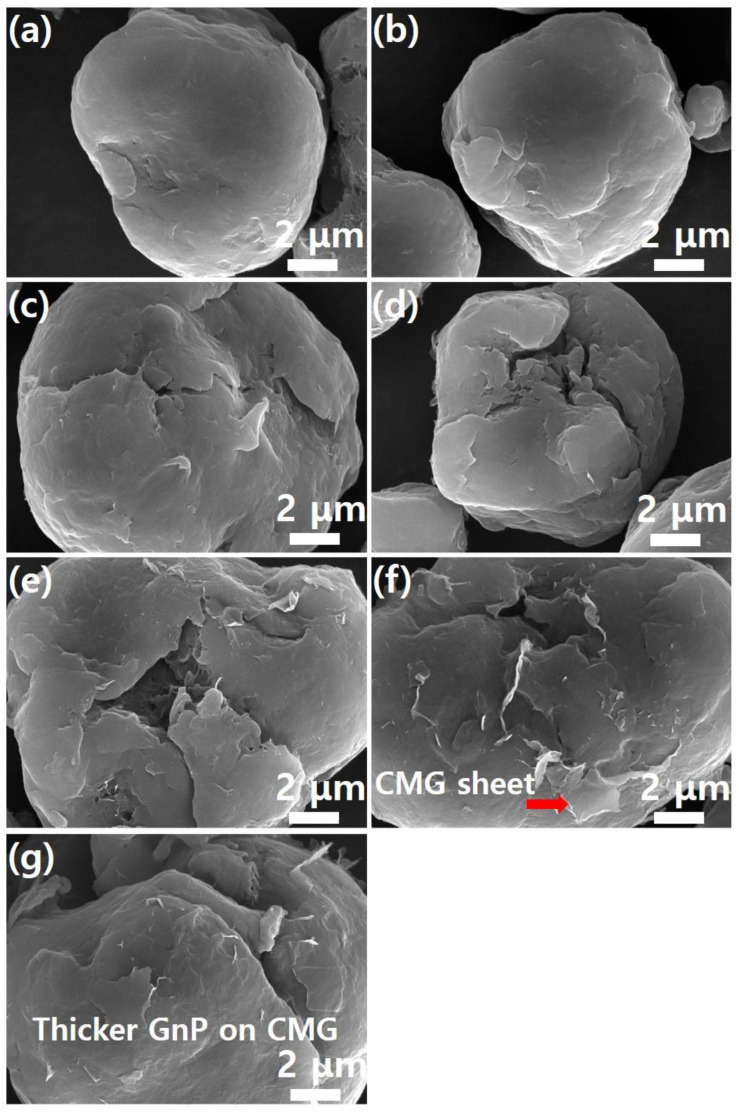
SEM images (high magnification) of PEEK–graphene powder for various graphene contents: (**a**) neat PEEK, (**b**) CMG0.1, (**c**) CMG0.3, (**d**) CMG0.5, (**e**) CMG1.0, (**f**) CMG2.0, and (**g**) CMG1.0/GnP1.0.

Meanwhile, as shown in Figure 4, pristine PEEK powder had a particle size of 20 μm or less. Furthermore, the graphene coating did not cause agglomeration of the PEEK powder. This suggests that the graphene coating did not physically damage the PEEK polymer. High-magnification SEM images confirmed the presence of the graphene coating on the PEEK surface. Figure 5 shows high-magnification (×20,000) SEM images of the images shown in Figure 4. As shown in Figure 5a, the surface of the PEEK powder was smooth. As the graphene content increased, the number of edge shapes (wrinkles and folds, which are unique morphological features of graphene) on the surface increased. This indicates the adsorption of the graphene sheet onto the PEEK surface. This phenomenon became more evident as graphene concentration increased. Figure 5f shows the morphology of the CMG sheet with a lateral size of 1–2 μm. As shown in Figure 4g, GnPs did not appear on the external surface of PEEK–graphene particles because they were physically adsorbed onto the CMG layer through van der Waals forces and π-π stacking. Furthermore, as shown in Figure 5g, the surface of the PEEK–graphene particles was enveloped by a thick GnP layer. Therefore, the analysis revealed that CMG^+^ in the PEEK–graphene nanocomposite formed a strong three-dimensional network through ion bonding, and GnPs could be easily detached from the surface of PEEK–graphene and dispersed freely into the GnP sheets inside the nanocomposites, supporting the hypothesis of this study.

**Figure 6:**
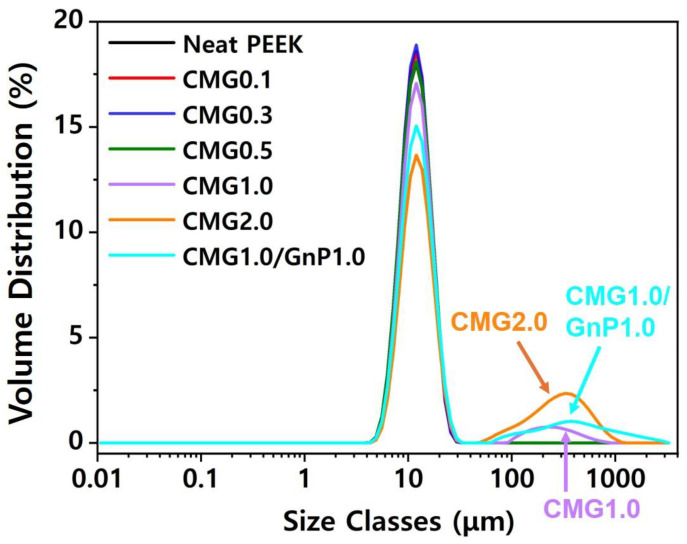
Particle size distributions in PEEK–graphene powder for various graphene contents.

Figure 6 and Figure S3 show the particle size distributions of the pristine PEEK and PEEK–graphene powders. Pristine PEEK had a uniformly dispersed distribution. When the graphene content was low, the particle distribution appeared to be uniform. However, when the graphene content exceeded 1.0 wt.%, particles larger than 100 μm could be observed. In particular, larger particles could be observed at a CMG content of 2.0 wt.%, compared to when the CMG content was 1.0 wt.%. This indicates that an increase in graphene content leads to the partial formation of interparticle networks. Particle size can be measured using various approaches. In this study, the particle size was determined based on the median value. This was defined as the value at which half of the population lies above it and the remaining half lies below it. The median particle size was referred to as D_v_50. The volume distribution was predominantly determined using laser diffraction, with the quoted baseline D50 representing the volume median. The pristine PEEK exhibited a D_v_50 of 11.8 μm. PEEK exhibited a D_v_50 of 11.7–11.8 μm when the CMG content was below 0.5 wt.%. When the CMG content was 1.0 and 2.0 wt.%, it exhibited a D_v_50 of 12.3 and 13.9 μm, respectively, indicating the generation of larger particles.

#### 3.2.2. Thermal Properties

As shown in Figure S2, the MI decreased with increasing graphene content. Each value represents the average of five measurements. Although error bars are included, the magnitudes of the errors are negligible, making them imperceptible on the graph. Pristine PEEK exhibited an MI of 72.0 g/10 min, indicating high flowability. In addition, the strand surfaces were smooth. However, when the concentration of graphene increased, the MI decreased in the order 58.1, 63.8, 54.1, 42.8, and 31.4 g/10 min. However, the surfaces of the strands became rougher as the graphene content increased, along with an increase in the thickness of the strands. CMG1.0/GnP1.0 exhibited an MI of 43.0 g/10 min, which implies that GnPs had minimal influence on flowability. However, the roughness of the strand surface increased with increasing GnP content, indicating that GnPs had a detrimental effect on the surface quality. The graphene coating layer was predicted to influence the flowability of PEEK–graphene powder. Moreover, graphene was expected to self-adsorb onto the surface of the PEEK powder, forming a core–shell structure (Figure 1). The pristine PEEK melted owing to heat, causing it to merge with the surrounding particles and form a single molten entity. The pristine PEEK melt exhibited high flowability as it was not hindered by obstacles. However, in the case of graphene-coated PEEK powder, the PEEK polymer, after melting, failed to come into contact with the surrounding polymer melt owing to the presence of graphene. This resulted in the formation of isolated domains that degrade the overall flow. To verify this, the crystallinity was examined using a polarization microscope.

**Figure 7:**
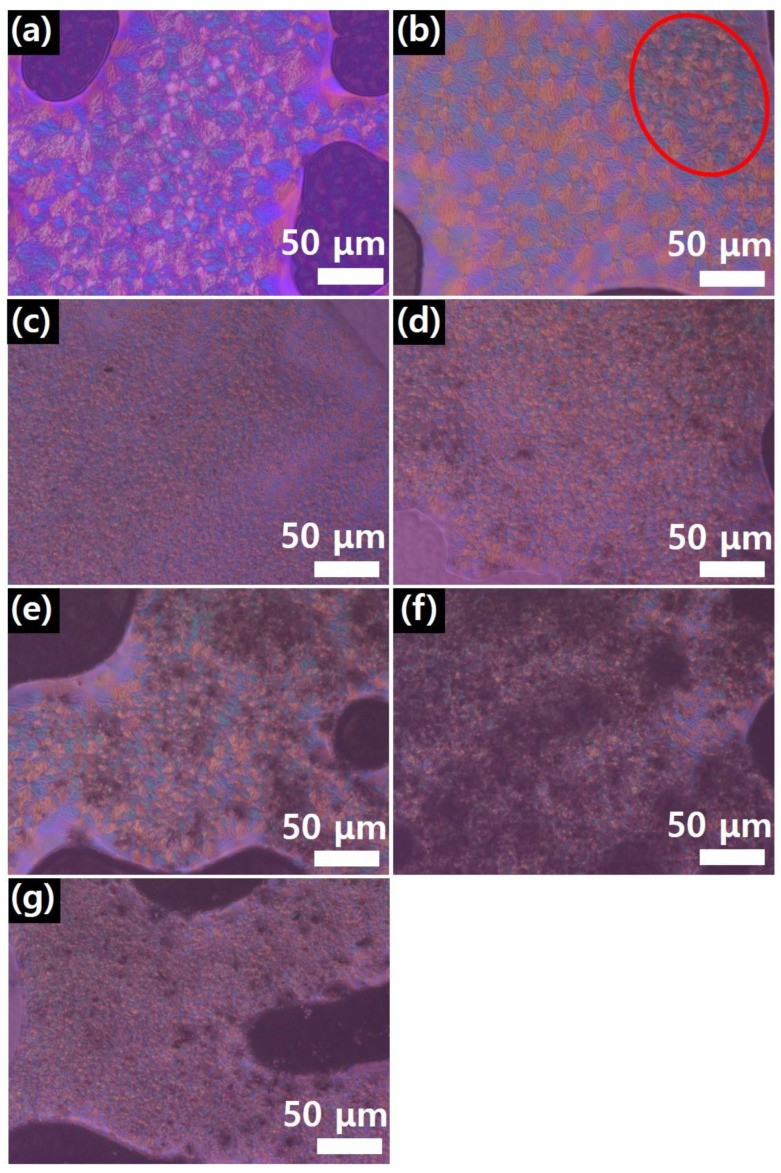
Polarization microscope image of PEEK–graphene powder for various graphene contents: (**a**) neat PEEK, (**b**) CMG0.1, (**c**) CMG0.3, (**d**) CMG0.5, (**e**) CMG1.0, (**f**) CMG2.0, and (**g**) CMG1.0/GnP1.0. The red circle in (**b**) highlights an area of uneven crystallization, indicating the influence of graphene on the crystallization behavior of PEEK.

Furthermore, Figure 7 shows the polarization microscopy images for various graphene contents. Figure 7a shows that neat PEEK had a uniform crystalline structure. CMG (0.1) exhibited an area of uneven crystallization (red circle). When the graphene content exceeded 0.3 wt.%, the size of PEEK crystals significantly decreased. Furthermore, a comparison of Figure 7e,g shows that the GnPs did not influence the formation of PEEK crystals. This trend is similar to the melt flow behavior of the PEEK–graphene powder.

**Figure 8:**
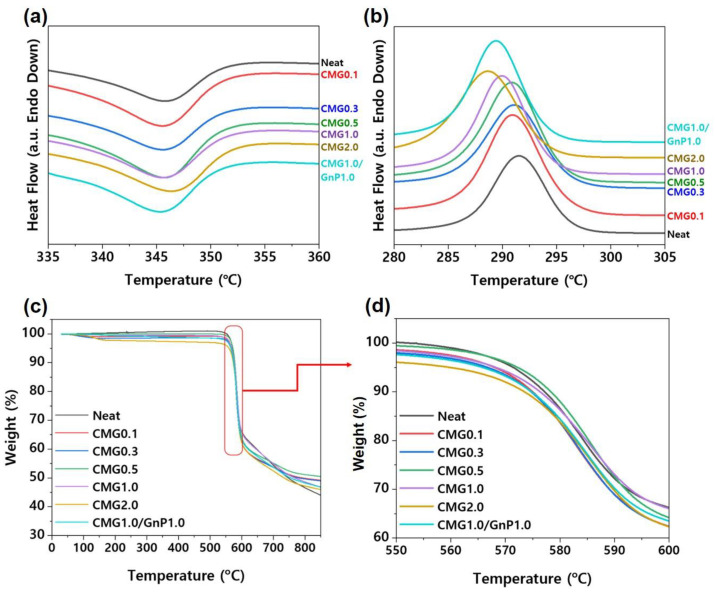
Non-isothermal DSC scan (**a**) heating curves and (**b**) cooling curves of PEEK–graphene nanocomposites for various graphene contents. (**c**) Thermogravimetric (TG) curves of PEEK–graphene nanocomposites for various graphene contents; (**d**) magnified view of the thermal degradation region, highlighting the effect of graphene content on the thermal stability and weight loss profile of the PEEK matrix.

To evaluate the influence of the nanofillers on the crystallization and melting behaviors of the PEEK matrix, a morphological study was conducted by analyzing the nanocomposites using DSC. The degree of crystallinity is known to significantly influence the mechanical properties of polymer materials. The blending of nanofillers can promote either nucleation or confinement, which are two independent effects that influence the crystallization of the polymer matrix. Nanofillers can provide heterogeneous sites to promote nucleation, thereby increasing the crystallization temperature [23]. However, the formation of a well-developed nanofiller network substantially restricts polymer chain diffusion and crystal growth, delays crystallization, and reduces yield [23]. Figure 8a,b shows the DSC curves for various graphene contents. The melting points of the nanocomposites were slightly lower than that of pristine PEEK during the heating scan. The lower T_m_ of the nanocomposites indicates the formation of smaller or less perfect crystals in the nanocomposite. Table S2 lists the values of T_m_, T_c_, crystallinity, and X_c_ obtained from DSC curves. According to the DSC results shown in Figure 8 and Table S2, the PEEK–graphene nanocomposites exhibited a higher degree of crystallinity than pure PEEK owing to the nucleating effect of graphene. Therefore, a lower T_m_ value of the nanocomposite material implies the formation of smaller crystals within it. This phenomenon is consistent with the observations derived from the polarization microscopy images of the samples (Figure 8).

The influence of graphene content on the thermal stability of the PEEK–graphene nanocomposites was analyzed using TGA. As shown in Figure 8c,d, pure PEEK exhibited a single decomposition stage under a nitrogen atmosphere. Decomposition included decarboxylation and dehydration reactions [23]. The decomposition of pure PEEK began at 572.6 °C, with the maximum rate observed at 585 °C (Table S3). The presence of CMG thermally destabilized the nanocomposites, owing to chemical interactions between PEEK and the decomposition products of CMG [21]. As shown in Figure 8d, the decomposition of the nanocomposites began at low temperatures. Char residues are formed at temperatures above 800 °C and are composed of carbonized solid residues with distinct aromatic structures. The influence of graphene content on the properties of the PEEK–graphene nanocomposites can be elucidated by examining T_d_ and T_max_. Graphene acts as a thermal stabilizer in polymers because it hinders the transition of volatile decomposition products from the bulk of the polymer to the gas phase [23,24]. At low concentrations, the presence of graphene does not affect the thermal stability of the nanocomposites. This suggests that the graphene dispersion is insufficient to promote a stabilizing effect. In contrast, the thermal stability of pure PEEK increased slightly when the graphene content exceeded 0.5 wt.%. Notably, the chemical modification of graphene was expected to have two contrasting effects on the thermal stability of composite materials. It enhanced the dispersion of the graphene sheet, thereby improving the thermal stability owing to a superior blocking effect. Compared with PEEK, low-molecular-weight functional groups were less thermally stable, consequently reducing the thermal stability of the PEEK composites. A comparison of the performances of CMG1.0/GnP1.0 with those of CMG1.0 and CMG2.0 revealed that GnPs slightly degraded the thermal stability. This implies that CMG1.0 and CMG2.0 exhibit higher thermal stability than the hybrid-type samples.

#### 3.2.3. Tribological Properties

**Figure 9:**
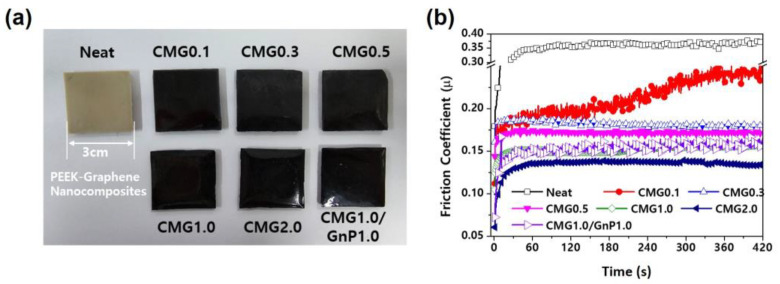
(**a**) Photographs of PEEK–graphene nanocomposites with different graphene contents. (**b**) Friction coefficient of PEEK–graphene nanocomposites as a function of time for various graphene loadings.

Figure 9 shows the friction coefficients of the PEEK–graphene nanocomposites as a function of graphene content. During the initial stages, the friction coefficient significantly increased owing to the strong frictional forces between the steel ball and the specimen. As the friction continued to increase, the two contact points gradually became slightly smoother, reducing the friction coefficient until it reached a stable stage. Pristine PEEK had a friction coefficient of approximately 0.35. The friction coefficient gradually decreased with increasing graphene content owing to the two-dimensional structural characteristics of graphene [22,23,24,25,26]. The friction coefficients of PEEK blended with CMG1.0 and CMG2.0 were approximately 54% and 63% lower than those of pure PEEK, respectively. Furthermore, compared with the CMG2.0 sample, the CMG1.0/GnP1.0 sample exhibited a higher friction coefficient. This can be attributed to the surface roughness of the strands, as observed in the MI measurement results. In the CMG1.0/GnP1.0 samples, the presence of GnPs deteriorated the surface quality and consequently had a negative influence on the friction behavior of the material. Puértolas et al. [1] and Kalin et al. [2] enhanced the friction properties of graphene-based PEEK nanocomposites; however, the graphene content exceeded 1 wt.% and the friction coefficient did not decrease by more than 40%. In this study, the friction coefficient was reduced by more than 50% by using low-loading graphene.

#### 3.2.4. Electrical Properties

**Figure 10:**
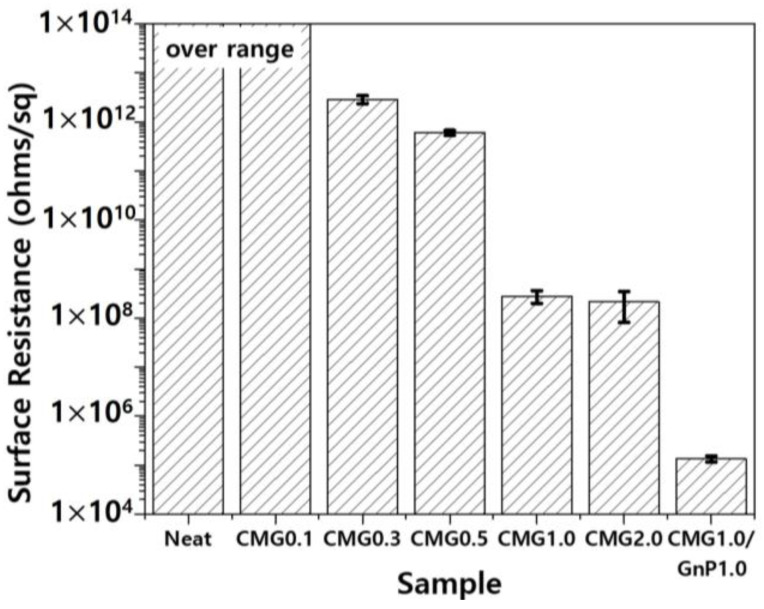
Surface resistance of PEEK–graphene nanocomposites for various graphene contents.

Electronic devices are vulnerable to electrostatic discharge, which is generated by frictional electrification during manufacturing, handling, packaging, and transportation. Electrostatic discharge can severely damage electronic equipment [17]. To mitigate electrostatic discharge, protective packaging with low electrical resistance is essential to prevent charge accumulation and facilitate charge flow [28]. Electrically insulating materials typically exhibit a relatively high surface resistance of at least 10^11^ ohms/sq, hindering the flow of electrons across the surface [19,20]. In contrast, materials with antistatic and dissipative properties exhibit an intermediate electrical resistance of 10^4^–10^8^ ohms/sq, which allows for electrical conduction [17,18]. Figure 10 shows the surface resistance of the PEEK–graphene nanocomposites as a function of graphene content. Pristine PEEK is an electrically insulating polymer, and its surface resistance exceeds the measurement range of the surface resistance meter used in this study. Hence, its surface resistance was 10^14^ ohms/sq or higher. Graphene was used to reduce the surface resistance of the PEEK–graphene nanocomposites. As shown in Figure 10, the CMG0.1 sample did not exhibit a decrease in surface resistance. However, CMG0.3 exhibited significantly reduced surface resistance in the range of 10^11^–10^12^ ohms/sq. The surface resistance of the nanocomposites gradually decreased with increasing concentrations of CMG^+^. At concentrations exceeding 1.0 wt.%, a further decrease was observed until reaching stability. As shown in Figure 10, the surface resistance of the CMG1.0/GnP1.0 sample was significantly lower than that of the CMG2.0 sample with the same graphene content. As confirmed by EA and Raman spectroscopy, the GnPs exhibited high crystallinity and purity. Hence, its electrical conductivity was higher than that of CMG^+^. However, to ensure that the nanocomposite exhibited adequate electrical performance, the additive required was higher than that of CMG^+^. This is because CMG^+^ was thicker, with a single layer measuring 1–2 nm. The excellent electrical performance of the CMG1.0/GnP1.0 provided the most convincing evidence that the CMG in PEEK–graphene forms a strong 3D network through ionic bonding. GnPs were easily desorbed from the surface of a PEEK–graphene nanocomposite and dispersed freely into graphene sheets inside the nanocomposite to form an optimal electrical pathway.

## 4. Conclusions

This study focused on enhancing the functionality of PEEK for semi-finished items. In particular, the wear particles of PEEK formed by frictional abrasion can significantly impact product defects, underscoring the importance of their anti-wear characteristics. In this study, functionalized graphene was self-adsorbed onto the surface of a PEEK powder to produce nanocomposites. In PEEK–graphene nanocomposites, graphene hindered the diffusion of polymer chains, reducing crystal size. In contrast, it improved the thermal stability of PEEK by impeding the movement of volatile decomposition products (barrier effect). In addition, graphene served as a solid lubricant, reducing the friction coefficient and surface resistance compared to pure PEEK. Graphene content of at least 1.0 wt.% was required to improve friction and electrical characteristics. However, the graphene content should be kept low considering the melt flow characteristics and size distribution. Therefore, an optimal adjustment of the graphene content in the nanocomposite is necessary to obtain the desired characteristics of the final product. The findings of this study are expected to enhance the utility of electrostatically dissipative PEEK. In future research, we plan to manufacture semi-finished products using PEEK–graphene nanocomposites and analyze their properties.

## Figures and Tables

**Table 1:** Normalized elemental compositions of GO, CMG^+^, and GnP.

	C (at.%)	O (at.%)	N (at.%)
GO	55.37	44.63	0
CMG^+^	80.10	9.85	10.05
GnP	99.87	0.12	0.01

## Data Availability

All data used during the study appear in the submitted article.

## Supplementary Materials

The following supporting information can be downloaded at: https://www.mdpi.com/article/10.3390/polym17060721/s1, Figure S1. SEM image of GnPs; Figure S2. MI of PEEK–graphene powders for various graphene contents; Figure S3. Histogram of the particle distributions for (a) Neat PEEK, (b) CMG0.1, (c) CMG0.3, (d) CMG0.5, (e) CMG1.0, (f) CMG2.0, and (g) CMG1.0/GnP1.0; Table S1. Elemental composition of GO, CMG+, and GnP. Measured and normalized weight percentages (wt.%) are presented alongside atomic percentages (at.%). Hydrogen-excluded normalized at.% values and C/O ratios are included for comparative analysis; Table S2. Summary of DSC analysis; Table S3. Summary of TGA analysis.
