# Supplementary material for: Enhanced Tribological and Electrical Performance of Graphene-Coated Polyetheretherketone Nanocomposites
Source: Polymers (Basel). 2025 Mar 9;17(6):721. doi: 10.3390/polym17060721 (PMC11944534; doi:10.3390/polym17060721)
Supplement: Supplementary file 1 [file polymers-17-00721-s001.zip › polymers-3482742-supplementary.pdf]

Supplementary information

Enhanced Tribological and Electrical Performance of Graphene-Coated Polyetheretherketone Nanocomposites

Pyoung-Chan Lee [1], Seo-Hwa Hong [1], Jung Hoon Kim [2], Jae Young Seo [2], Youn Ki Ko [1], Jin Uk Ha [1], Sun Kyoung Jeoung [1], Myeong-Gi Kim [2,*], Beom-Gon Cho [3,*]

[1] Chassis & Materials Research Laboratory, Korea Automotive Technology Institute, 303 Pungse-ro, Pungse-myeon, Dongnam-gu, Cheonan-si, Chungcheongnam-do 31214, Republic of Korea; pclee@katech.re.kr (P.-C.L.); shhong1@katech.re.kr (S.H.H); ykko@katech.re.kr (Y.K.K); juha@katech.re.kr (J.U.H); skjeong@katech.re.kr (S.K.J)
[2] R&D Center, BESTGRAPHENE Co., Ltd, Yeoju-si, Gyeonggi-do 12616, Republic of Korea; run8827@best-graphene.com (J.H.K); jyseo@best-graphene.com (J.Y.S); mgkim@best-graphene.com (M.-G.K);
[3] Department of Polymer Science and Engineering, Kumoh National Institute of Technology, 61 Daehak-ro, Gumi, Gyeongbuk 39177, Republic of Korea
bgcho@kumoh.ac.kr (B.-G.C)

*Correspondence: mgkim@best-graphene.com (M.-G.K) Tel.: (+82-31-883+8858); bgcho@kumoh.ac.kr (B.-G.C) Tel.: (+82-54-478-7684)

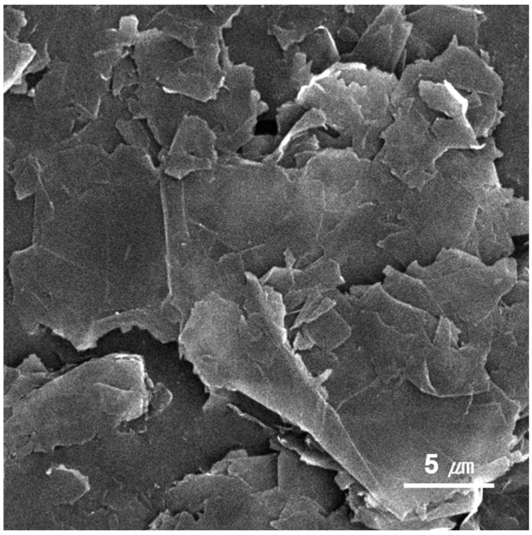

Figure S1. SEM image of GnPs.

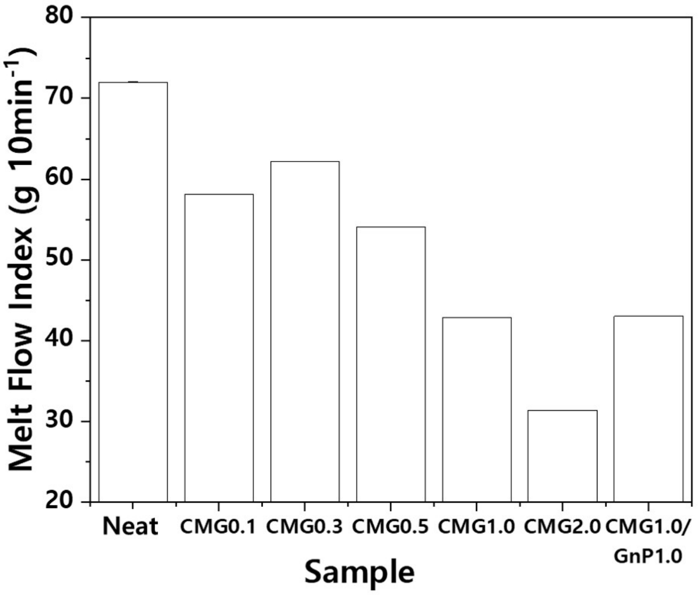

Figure S2. MI of PEEK–graphene powders for various graphene contents.

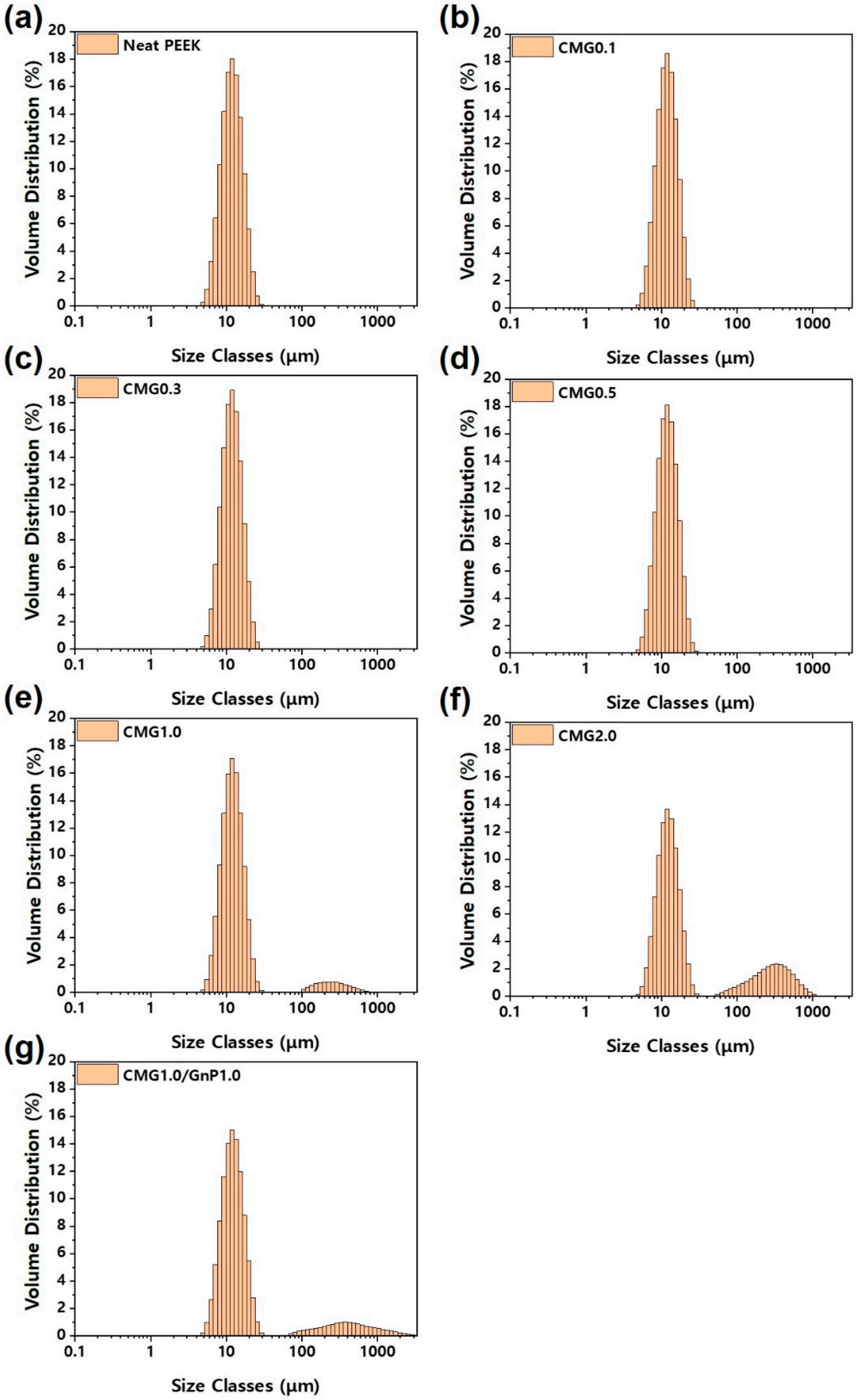

Figure S3. Histogram of the particle distributions for (a) Neat PEEK, (b) CMG0.1, (c) CMG0.3, (d) CMG0.5, (e) CMG1.0, (f) CMG2.0, and (g) CMG1.0/GnP1.0.

Table S1. Elemental composition of GO, CMG+, and GnP. Measured and normalized weight percentages (wt.%) are presented alongside atomic percentages (at.%). Hydrogen-excluded normalized at.% values and C/O ratios are included for comparative analysis.

	Measured wt.%					Normalized wt.%					Normalized at. %					Normalized at. %				
Type	C	H	O	N	total	C	H	O	N	total	C	H	O	N	total	C	O	N	total	C/O ratio
GO	40.83	2.71	43.85	0	87.4	46.73	3.10	50.18	0.00	100.0	38.52	30.43	31.05	0.00	100	55.37	44.63	0.00	100	1.240
CMG+	72.88	3.52	11.94	10.66	99.0	73.62	3.56	12.06	10.77	100.0	54.83	31.55	6.74	6.88	100	80.10	9.85	10.05	100	8.131
GnP	92.78	0.25	0.15	0.01	93.2	99.56	0.27	0.16	0.01	100.0	96.77	3.11	0.12	0.01	100	99.87	0.12	0.01	100	823.904

Table S2. Summary of DSC analysis.

Sample	T_m (°C)	T_c (°C)	X_c (%)
REF	342.02	295.04	26.79
CMG0.1	341.84	294.72	35.84
CMG0.3	341.85	294.67	30.51
CMG0.5	341.97	294.54	33.28
CMG1.0	341.83	293.70	32.43
CMG2.0	342.66	292.36	32.41
CMG1.0/GnP1.0	341.49	293.04	31.42

Table S3. Summary of TGA analysis.

Sample	5 % weight loss temperature (°C)	T_{D1} (°C)	Residue (%)
REF	559.27	572.6	43.91
CMG0.1	556.33	572.3	49.19
CMG0.3	554.58	572.4	48.92
CMG0.5	560.30	575.1	50.49
CMG1.0	556.52	574.5	46.81
CMG2.0	547.86	574.7	45.92
CMG1.0/GnP1.0	554.48	573.9	43.59
